# Impact of methamphetamine on infection and immunity

**DOI:** 10.3389/fnins.2014.00445

**Published:** 2015-01-12

**Authors:** Sergio A. Salamanca, Edra E. Sorrentino, Joshua D. Nosanchuk, Luis R. Martinez

**Affiliations:** ^1^Department of Biomedical Sciences, Long Island University-PostBrookville, NY, USA; ^2^Microbiology and Immunology, Albert Einstein College of MedicineBronx, NY, USA; ^3^Medicine (Division of Infectious Diseases), Albert Einstein College of MedicineBronx, NY, USA; ^4^Department of Biomedical Sciences, NYIT College of Osteopathic Medicine, New York Institute of TechnologyOld Westbury, NY, USA

**Keywords:** methamphetamine, infectious diseases, immunity, drug abuse, HIV, neurotoxicity

## Abstract

The prevalence of methamphetamine (METH) use is estimated at ~35 million people worldwide, with over 10 million users in the United States. METH use elicits a myriad of social consequences and the behavioral impact of the drug is well understood. However, new information has recently emerged detailing the devastating effects of METH on host immunity, increasing the acquisition of diverse pathogens and exacerbating the severity of disease. These outcomes manifest as modifications in protective physical and chemical defenses, pro-inflammatory responses, and the induction of oxidative stress pathways. Through these processes, significant neurotoxicities arise, and, as such, chronic abusers with these conditions are at a higher risk for heightened consequences. METH use also influences the adaptive immune response, permitting the unrestrained development of opportunistic diseases. In this review, we discuss recent literature addressing the impact of METH on infection and immunity, and identify areas ripe for future investigation.

## Methamphetamine (METH), a major public health problem

The growing popularity of Methamphetamine (METH), a street drug associated with the severe neurological and physical consequences afflicting its users, has created an increasingly serious public health problem worldwide. In a 2011 United Nations survey, approximately 2.5% of Australians over the age of 14 have tried METH, a prevalence rate three to five times higher than those seen in the United States (USA), Canada, and the United Kingdom (United Nations, 2011). In the USA, over one million individuals aged 12 years and older—roughly 0.5% of the American population—were reported to have sampled METH (Colfax and Shoptaw, 2005; United Nations, 2011). According to the USA Department of Justice, after alcohol and marijuana, METH is the most commonly used recreational drug in many states (Drug Enforcement Administration, 2007).

METH is a potent central nervous system (CNS) stimulant that mimics the pharmacological effects of cocaine. The “rush” that follows METH use is associated with the release of neurotransmitters, including adrenaline, dopamine, and serotonin (Downes and Whyte, 2005; Collins et al., 2014). Whereas the half-life of cocaine is measured in minutes, however, that of METH is measured in hours (~8 to 24 h). Thus, the pharmacological effects of METH are thus longer lasting than cocaine. In spite of the potentially dangerous consequences of METH use, the drug retains its popularity as a low-cost alternative to cocaine and heroin. The relative ease of METH production has ensured that prices remain low, particularly in Australia, where METH use is more prevalent and widespread than in most other countries (Marwick, 2000; United Nations, 2011; Gong et al., 2012). In recent years, however, the upsurge of drug enforcement and control policies has significantly limited the availability of precursor chemicals essential for METH production, raising purchase prices and reducing the overall demand for the product (Drug Enforcement Administration, 2007; Gong et al., 2012). General METH use is seen as minimal exposure to the drug, primarily involving first time users; whereas, chronic METH abuse and dependence expose the user to a diverse range of adverse physical and cognitive health consequences (Panenka et al., 2013). The rate of treatment admissions for primary METH abuse has increased over 3-fold in recent years (Colfax and Shoptaw, 2005).

Diverse routes for METH use exist, including oral ingestion, smoking, snorting, intravenous injection, and anal insertion. The intravenous administration of METH has become a popular usage mechanism due to its ability to deliver almost immediate effects of euphoria (Hart et al., 2008). The sharing of drug paraphernalia combined with METH's perceived enhancement of sexual pleasure and the association of its use with unsafe sexual practices greatly increases the likelihood of the acquisition of human immunodeficiency virus (HIV) and other infectious diseases (Ellis et al., 2003; Urbina et al., 2004; Mansergh et al., 2006; Nakamura et al., 2011). In addition, animal studies demonstrate that METH suppresses both innate and adaptive immunity (In et al., 2005; Peerzada et al., 2013). This review explores recent research developments related to the effects of METH on infection and immunity.

## Pharmacological METH levels in humans

The S-(+) enantiomer of METH ((S)-N-Methyl-1-phenyl-propan-2-amine), dextromethamphetamine, is popularly used among METH users for its potent effect on the cardiovascular system and CNS (Li et al., 2010; Volkow et al., 2010). Patterns of METH intake are variable depending on the user; a self-reporting study indicated that a majority of chronic METH users consume the drug more than 20 days per month, at a frequency of 1–3 doses per day (Saito et al., 2008). Typically, people take 5–15 mg (low stimulation), 10–30 (common dose), and 20–60 mg (strong) with both per-oral and intravenous (i.v.) administration (Hart et al., 2008; Cruickshank and Dyer, 2009). Following ingestion, the metabolism of METH takes place in the liver, where the cytochrome P4502D6 causes N-demethylation and aromatic hydroxylation, forming the primary metabolites para-hydroxymethamphetamine (pOH-MA) and amphetamine (AMP). Afterwards, the primary and other minor metabolites (norephedrine, 4-hydroxyamphetamine, 4-hydroxynorephenedrine, benzyl methyl ketoxime and benzoic acid) are absorbed across the gastrointestinal tract. The concentration peak of METH in plasma after oral ingestion can be detected at 3.13–6.3 h post-consumption and its metabolites peak at 10–24 h (Gartner and Liu, 2002). The metabolite pOH-MA is therefore one of the most stable biomarkers of METH abuse (Li et al., 2010).

METH is often used in binges, and as the drug exhibits a half-life of 11.4–12 h (Cho et al., 2001; Harris et al., 2003). Recently published studies modeling binge patterns show that after the fourth administration of 260 mg during a single day, subsequently, produces blood levels of 2.5 mg/L, reaching as high as 3 mg/L on the second day (Melega et al., 2007). Thus, binge doses of 260 mg–1 g produce 2.5–12 mg/L blood levels. A study conducted in Australia between 2000 and 2005 found that 68% of 371 deaths in which individuals tested positive for AMPs could be attributed directly to METH toxicity. METH concentration ranged from 0.2 to 15 mg/L (median, 0.2 mg/L), with AMP levels registering at 0.01–2.0 mg/L (median, 0.07 mg/L) (Kaye et al., 2008). It is important to establish that these concentrations and peak values vary greatly depending upon the routes of administration and detection technique.

Although the brain receives around 15% of the cardiac output (114 ± 24 mL/100 mL/min) the concentration of METH, its distribution and metabolism varied in all the organs (Ito et al., 2003). Interestingly, the effect of METH in brain structure and activity is extensive. A study determined the distribution and bioavailability of METH in several human organs using Positron Emission Tomography, revealing a low rate of drug uptake in the brain (9 min) compared to the other organs examined. Nevertheless, the prolonged clearing period (>75 min), suggests a neurotoxic effect due to the extended exposure to the drug (Volkow et al., 2010).

## Pharmacological METH levels in animals

The use of animal models has been widely used to evaluate the effect of METH in the immune and nervous system, among others. One study employing a murine model estimated how METH is distributed to tissues. Tissue-to-serum METH ratios in rats are: brain, 9.7; kidney, 35.3; spleen, 14.3 (Rivière et al., 2000). Levels of METH and AMP in both female and male murine spleens measured within a 72 h period after treatment with 5 mg/Kg demonstrated high concentrations of METH (Male, 870, Female, 1310 ng/g) in comparison to lower levels of AMP (Male, 130, Female, 270 ng/g) within the first hours after the initial injection (Saito et al., 2008). However, a typical dose of METH that is self-administered (i.v.) by rats is 0.1–0.2 mg/kg that is equivalent to a human dose (7–14 mg/70 kg) (Cook et al., 1992; Hart et al., 2008; Kuczenski et al., 2009; Krasnova et al., 2010; Kousik et al., 2014). Also, pigeons injected i.v. and intramuscularly (i.m.) with 0.8 mg/kg of METH showed 100% of bioavailability; however, i.v. absorption was three times higher than i.m. In this regard, some studies support that injection of METH in ranges of 3.6–10 mg/Kg are considered lethal in animals (Hendrickson et al., 2008). Similarly, a recent study showed an 8-fold higher catalytic activity of METH in rhesus macaques compared to humans due to enzymatic differences (Earla et al., 2014).

## Mechanisms of neurotoxicity

Administration of METH can increase blood–brain barrier (BBB) permeability in rodents. Moderate to high doses of METH disrupt the BBB in several regions, including the cortex, hippocampus, thalamus, hypothalamus, cerebellum, amygdala, and striatum that, in turn, are further injured by hyperthermia and, potentially, by seizures (Sharma and Kiyatkin, 2009; Yamamoto et al., 2010). Although it is unclear whether there is a relationship between BBB injury and the damage to neurotransmitter systems, BBB injury appears to contribute to striatal neuron degeneration rather than dopaminergic terminal damage (Bowyer et al., 2008). METH stimulates astrocytes to produce high levels of IL-6 and IL-8, resulting in an inflammatory response that inhibits neurogenesis in the brain, affects sub-ventricular and hippocampal cells, reduces hippocampal progenitor cells. Similarly, METH alters gene expression on astrocytes, halting their cell cycle and proliferation (Shah et al., 2012; Jackson et al., 2014).

Mechanisms underlying METH-induced BBB damage include alterations of expression and structure in tight junctions, microglial activation, remodeling of BBB cytoskeleton, induction of neuroinflammatory factors, and energy related disruption. METH, especially at high doses combined with physical exertion, can cause hyperthermia and enhance reactive oxygen species (ROS) production, thus triggering BBB breakdown (Sharma et al., 2007; Ramirez et al., 2009; Northrop and Yamamoto, 2012). METH can induce the polymerization of proteins necessary for the stability of the BBB. Therefore, when alterations in proteins occur, the permeability of the barrier is affected and migration of inflammatory cells, such as monocytes, arises more frequently (Park et al., 2013). In murine models, the administration of low and high doses of METH shows an increase in IgG immunoreactivity in the striatum (Urrutia et al., 2013).

Notably, administration of antioxidants attenuates BBB injury in acute METH toxicity models and further implicates oxidative stress in pathological effects (Sharma et al., 2007). One study suggests that METH induces the opening of the BBB by activating the nitric oxide synthases (NOS) present in the endothelial cells of the brain capillary network (Martins et al., 2013). Oxidative stress represents an imbalance between the production of ROS and the BBB's ability to readily detoxify the reactive intermediates or to repair the resulting damage.

METH also alters the expression of several tight junction proteins and increases the permeability of brain-derived primary microvascular endothelial cells (Mahajan et al., 2008; Ramirez et al., 2009). The use of acute high doses of METH increases the permeability of the BBB principally in the hippocampus, while downregulation of tight junction proteins such as ZO-1, Claudin-5, and occludin cause failure in the BBB, thus increasing the expression of matrix metalloproteinase (MMP)-9 in the hippocampal neurons (Martins et al., 2011). The regulation of occludin levels is important to maintain the stability of the endothelial tissue; however, METH causes the polymerization of actin thereby hindering rearrangements, ultimately leading to a functional disruption of the BBB (Park et al., 2013). MMP activation is thought to occur through several mechanisms, including oxidative stress and cytokine production (Haorah et al., 2007; McColl et al., 2008).

Collectively, these findings suggest that AMP-driven oxidative stress followed by the activation of MMPs and breakdown of tight junctions mediate BBB disruption; both the activation of MMPs and oxidative stress can induce inflammation which could be accompanied by an increase in cytokine production within microglia, perpetuating damage and increasing BBB permeability (Kim et al., 2005; Amantea et al., 2007; Block and Hong, 2007). The consequences of BBB disruption are widespread and may enhance the vulnerability of the brain to microbial toxins and infection (Eugenin et al., 2013).

## Effects of METH on host immunity

The effects of METH on host immune response have not yet been extensively described. Limited studies about the effects of METH on immune function have, however, revealed that METH use has profound immunological implications. Findings in humans, with slight variance across ethnic groups, reveal that the uptake of a particular METH isotope targets specific organ types, in which concentrations (per/mL of tissue) were highest in the kidneys and lungs; intermediate in the stomach, pancreas, liver, and spleen; and lower in the brain and heart (Volkow et al., 2010). METH use leads to profound consequences in both, innate and adaptive immunity. Hence, investigations have begun to further elucidate the cellular and molecular basis for METH's induced immune suppression, examples of which are discussed subsequently.

### METH alterations of natural physical and chemical barriers

The skin acts as a primary physical barrier to prevent the entrance of pathogens, thereby serving as one of the innate immune response's first lines of defense (Proksch et al., 2008). Sweat glands in the skin release various bactericidal and regulatory peptides, restricting the development of pathogenic microbiota (Rieg et al., 2006). METH has been detected in sweat 2 h after ingestion, with traces remaining for periods of more than a week in cases wherein multiple doses were administered (Barnes et al., 2008). No previous studies exist, however, aiming to understand the effect of METH on microbiota and metabolites present in the skin (e.g., lactate, glycerol, pyruvate, ammonium cation, urea) (Kutyshenko et al., 2011). In this regard, the administration of drugs such as METH via injection is associated with the development of necrotizing fasciitis. Significantly, heavy daily users of METH frequently develop neurological manifestation of formication, a sensation akin to insects crawling on or under the skin. The result of formication is that users engage in constant skin “picking,” often causing the formation of ulcers that frequently scar. A marked lack of hygiene among users may also be correlated to higher rates of skin infections, abscess, and cellulitis (Rusyniak, 2013).

Another common sign of METH abuse is extreme tooth decay, a condition known in the media as “METH mouth.” Users with “METH mouth” have blackened, stained, or rotting teeth, even among young and/or short-term users. The exact causes of “METH mouth” are not fully understood. A common misconception is that METH directly causes the caries (Shaner et al., 2006). The leading hypothesis is that METH constricts blood vessels, thereby, limiting blood supply resulting in “dry mouth” or xerostomia (Saini et al., 2005; Goodchild and Donaldson, 2007; Heng et al., 2008; Hamamoto and Rhodus, 2009). A reduction in saliva impairs the mouth's capacity to neutralize harsh acids produced by oral bacteria after metabolizing carbohydrates, resulting in erosion of the teeth and gums and increasing the susceptibility of teeth to damage (Shaner et al., 2006; Evans et al., 2012). A more recent pilot study, however, found no difference in saliva flow rates between users and non-users despite increased saliva acidity in users and decreased buffer capacity in saliva.

The extent of tooth decay varies widely among METH users. Richards et al., found that users who snorted METH had significantly worse tooth decay than users who smoked or injected it, although all types of users suffered from dental problems (Richards and Brofeldt, 2000); however, a newer study suggests the oral route, in contrast to intravenous or intranasal, as a better predictor of “METH mouth” severity (Brown et al., 2013).

### Role of METH on innate immunity

METH administration induces modifications in cellular components including natural killer cells (NK), dendritic cells (DCs), monocytes, macrophages, and granulocytes, indicating complex mechanisms of immunosuppression (Harms et al., 2012). METH alkalizes normally acidic organelles within macrophages, leading to the inhibition of phagocytosis and antigen presentation processes (Tallóczy et al., 2008). Similar to chloroquine, METH is a weak base capable of inducing a collapse of the pH gradient across acidic organelles. The microbicidal capacity of DCs and macrophages is significantly decreased after METH exposures (Tallóczy et al., 2008; Martinez et al., 2009). Furthermore, the drug reduces the number of DCs and NK cells (Saito et al., 2006; Harms et al., 2012). The reduction of monocytes (Harms et al., 2012) and macrophages in the peritoneal zone after METH administration has also been reported (Saito et al., 2008). Similarly, antigen presentation in professional phagocytes are dysregulated, diminishing the processing capacity of these cells (Harms et al., 2012). METH-treated macrophages in tissue culture displayed increased levels of pro-inflammatory cytokine TNF-α, whereas similar cells stimulated with lipopolysaccharide (LPS) showed increased amounts of IL-1β and IL-8 in addition to TNF-α (Liu et al., 2012). These modifications of the innate immune response can result in impaired inflammatory responses and the degradation of physical and chemical protective barriers.

### METH and inflammation

Much of the existing literature related to METH's impact on inflammation derives from research focusing on CNS toxicity. For instance, METH increases glutamate (GLU) levels (Ito et al., 2006) and GLU receptor stimulation increases microglial activation (Thomas and Kuhn, 2005). Activation of GLU receptors increases the production of TNF-α, IL-1β, IL-6, and IL-8 (Chaparro-Huerta et al., 2005; Liu et al., 2012), resulting in increased extracellular GLU levels by either inhibiting GLU uptake or increasing GLU release from activated microglia (Zou and Crews, 2005). Additionally, astrocytes play a role in METH-induced toxicity through the modulation of GLU-mediated excitotoxicity and inflammation. Astrocytes regulate extracellular concentrations of GLU, mainly via neurotransmitter uptake. For METH, the activation of cortical astrocytes appears to be caused by GLU release and protein kinase C activation, and is inhibited by GLU receptor antagonism (Miyatake et al., 2005). Moreover, METH's stimulation of excitatory neurotransmitters and subsequent mGluR5-mediated activation of Akt/PI3K signaling pathways leads to the release of NF-kB, which then translocates from the cytoplasm to the nucleus for the enhanced expression of IL-6 and IL-8 in astrocytes (Shah et al., 2012). The release of NF-kB into the cytoplasm occurs via the phosphorylation of IKK by activated Akt/PI3K, which subsequently phosphorylates p-IkB, a regulatory protein for NF-kB (Shah et al., 2012). Under normal physiologic conditions, however, astrocytes suppress microglial activation through the release of anti-inflammatory cytokines and neurotrophic factors (Neumann, 2001). For instance, astrocytes suppress microglial activation by releasing TGF-β or IL-10 (Loftis et al., 2011).

Another mechanism by which METH facilitates inflammatory response is through the induction of oxidative stress. METH administration stimulates a substantial production of dopamine and the release of serotonin, which can undergo autoxidation processes and produce hydrogen peroxide and super-oxide radicals (Flora et al., 2003). In addition, METH can intensify cellular oxidation via the depolarization of mitochondria and, as mentioned previously, enhanced production of extracellular GLU, both of which are well known to boost levels of ROS (Shah et al., 2012). These oxidative disturbances in cellular redox status can incite the activation of various transcription factors, such as NF-kB, AP-1 or CREB, which, in turn, stimulate specific redox-regulated transcription factors that regulate gene expression for inflammatory cytokines and adhesion molecules (Shah et al., 2012).

### METH and adaptive immunity

T-cells play critical roles in orchestrating immune responses (Anderton, 2006) because their activation and proliferation are characteristic of adaptive immune responses. The mechanisms underlying the interplay between cells of the adaptive immune system and METH are currently unclear. However, the data firmly establishes that METH adversely impacts adaptive responses that render the host more susceptible to progressive diseases, particularly HIV (In et al., 2005; Martinez et al., 2009).

Murine models show that METH modifies thymic and splenic cellularity and alters peripheral T lymphocyte populations (In et al., 2005). High dose METH intake induces apoptotic death in rat thymic and splenic lymphocytes and produces severe immunosuppression, which could contribute to the higher rate of infections observed in chronic METH users (Harms et al., 2012; Peerzada et al., 2013). For instance, rodent studies demonstrate that METH alters cytokine response in retroviral-infections (Yu et al., 2002; Liang et al., 2008), alters gene expression of immune cells (Mahajan et al., 2006), and disturbs thymic CD4^+^/CD8^+^ T-cell ratios (Yu et al., 2002; In et al., 2005).

METH reduces T cell infiltrates in the lungs, inhibiting T cell proliferation and reducing the capacity of these cells to maintain a protective immune response against respiratory pathogens (Martinez et al., 2009). Similarly, METH-exposed mice demonstrated elevated levels of early response IL-6 and IL-10 in tissue homogenates, which could indicate the development of a non-protective Th2 response against bacterial and fungal pathogens in the respiratory tract, even when Th1 cytokines are present (Peerzada et al., 2013).

An alternative mechanism for altered T-cell function is that METH modifies oxidative stress responses. As discussed earlier, the effects of oxidative stress on suppressed signal transduction, transcription factor activities, and diminished cytokine production in response to antigen stimulation in T cells has been documented in several model systems (Flora et al., 2003; Shah et al., 2012). The ability of reactive oxidative free radicals to impair T lymphocyte function has been documented in various human pathologic conditions, specifically AIDS, in which oxidative stress can hamper host control of retroviral replication (Potula et al., 2010).

Interestingly, a recent finding suggests that METH alters intracellular calcium mobilization in T cells, resulting in subsequent production of oxidative free radicals, a phenomenon associated with mitochondrial damage and weakened T cell function (Potula et al., 2010). Mitochondria serve as a source of both intracellular ROS and ATP production, a process regulated by the second messenger, calcium. METH exposure elevates levels of cytosolic calcium, however, and leads to the saturation of the electron transport chain, which contributes to the acute production of oxidative free radicals and ultimately results in oxidative alteration of proteins, loss of intracellular ATP levels in T cells and mitochondrial dysfunction (Potula et al., 2010). A compensatory down-regulation of mitochondrial proteins from chronic METH treatment can incite a long-term cellular redox imbalance, weakening T cells' ability to effectively respond to opportunistic pathogens (Potula et al., 2010; Chandramani Shivalingappa, 2012; Martins et al., 2013).

## METH facilitates the acquisition of infectious diseases

In addition to psychosocial aberrations, infections are serious complications of chronic METH use. Moreover, the intoxicating effects of METH alter judgment and reduce inhibitions, leading people to engage in unsafe activities, increasing risk for acquiring transmissible microbes and other opportunistic infections; these findings have been documented worldwide (Plankey et al., 2007; Volkow et al., 2007; Ye et al., 2008; Sutcliffe et al., 2009; Parry et al., 2011; Borders et al., 2013; Eugenin et al., 2013; Heninger and Collins, 2013; Khan et al., 2013; Stahlman et al., 2013; Liao et al., 2014). Former and current drug users have higher risks to acquired sexually transmitted diseases (STDs) (Barry et al., 2009; Miller et al., 2009; Cranston et al., 2012; Javanbakht et al., 2012; Wang et al., 2012; Chew Ng et al., 2013). These infections result from the high association of METH use and inconsistent condom use, unprotected sex incentivized by money, and high-risk sexual partner types (Johnston et al., 2010; Borders et al., 2013; Stahlman et al., 2013). Hence, there are increased risks for diverse infectious diseases and these impaired individuals have a reduced capacity to combat microbial challenges (Cohen et al., 2007; Patel et al., 2013). In this regard, current clinical and empirical knowledge on the impact of METH on the acquisition of infectious diseases is discussed here.

### Methicillin-resistant Staphylococcus aureus (MRSA)

MR*SA* is the single most important bacterial pathogen in infections among injection drug users, with skin and soft-tissue infections (SSTI) being extremely common (Gordon and Lowy, 2005). Their incidence is difficult to estimate because such infections are often self-treated. In this regard, a study revealed that MR*SA* was isolated from 61% of abscesses and 53% of purulent wounds evaluated in the US emergency departments in all type of patients suggesting that it is likely that complicated cutaneous lesions in drug users are caused by this bacterium A cross-sectional study of IDUs in San Francisco found that 32% had an abscess, cellulitis, or both (Binswanger et al., 2000). Nasal carriage of MR*SA* is significantly increased in METH uses and MR*SA* disease occurs in over half of colonized drug addicts (El-Sharif and Ashour, 2008).

In addition, skin-picking is also associated with MR*SA* SSTI. As previously stated, METH use causes formication, which can lead to skin-picking behavior and skin breakdown. METH abusers often live in unhygienic circumstances. Moreover, unsafe injection of METH and poor injection hygiene (e.g., lack of skin cleaning before injecting), injecting with unsterile equipment and contaminated drug solutions can introduce high bacterial loads (Frontera and Gradon, 2000). Significantly, drug solutions may contain particulate matter (e.g., talc) that damage cardiac valves if injected intravenously (Frontera and Gradon, 2000). Chronic METH use may increase the incidence of cardiovascular pathology (Wijetunga et al., 2003; Yu et al., 2003) and, if injected, infective staphylococcal endocarditis (Cooper et al., 2007).

### STDs

The mind-altering effects of METH cause behavioral modifications, leading people to engage in sexual activities that put them at risk for acquiring transmissible diseases (Ellis et al., 2003). In addition to HIV and hepatitis, METH use is associated with an increased risk for and incidence of other STDs, including genital warts, syphilis, gonorrhea, and chlamydia (Hirshfield et al., 2004a,b; Mansergh et al., 2006; Rhodes et al., 2007; Mimiaga et al., 2008; Barry et al., 2009; Cranston et al., 2012; Javanbakht et al., 2012; Valencia et al., 2012). In a USA study, bacterial and viral STDs were significantly more common in METH users (odds ratio 3.8), and the risk to acquire STDs in METH users was even greater than that associated with cocaine (Hirshfield et al., 2004b). Furthermore, high levels of METH use are observed in a poly-drug use lifestyle, raising sexual risky behaviors (Khan et al., 2013). In particular, METH use is associated with increased risk for syphilis and gonorrhea in gay and bisexual men (Shoptaw et al., 2002; Wong et al., 2005; Taylor et al., 2007). In this regard, METH use is associated with the syphilis cases reported in China, including heterosexual and homosexual men and female sex workers (Kang et al., 2011; Liao et al., 2013, 2014).Furthermore, syphilis infection increases the transmission and acquisition of HIV (Xiao et al., 2010). The minimal amount of studies aiming to address the correlation between METH use and syphilis cases in several countries may dampen what role this drug plays in disease transmission and resistance to antibiotics.

### Hepatitis

METH abuse, hepatitis C virus (HCV) infection and HIV disease are overlapping epidemics in the USA and worldwide (Soriano et al., 2002; Letendre et al., 2005). Illicit drug-using individuals are at especially high risk for acquisition of and disease from HCV (Day et al., 2003; Hagan et al., 2005; Smyth et al., 2005). HCV results in ~20,000 infections and 8000–10,000 deaths annually in the USA (Ye et al., 2008; Klevens et al., 2009). HCV infection is particularly associated with injection use (Gonzales et al., 2006). Notably, HCV is prevalent in HIV patients (Ranger et al., 1991). In fact, HIV-HCV co-infection is found in 50–90% of HIV-infected drug users and chronic HCV infection increases the morbidity and mortality rates (Letendre et al., 2005; Soriano et al., 2002). Hence, a substantial proportion of METH users with or without HIV infection has HCV (Hahn et al., 2001; Miller et al., 2004; Lea et al., 2013), suggesting that METH abuse is a risk factor for HCV. Importantly, METH abuse significantly increases HCV penetration into the brain of HIV-infected patients, exacerbating cognitive impairments (Letendre et al., 2007). Although risky behavioral practices, such as sharing contaminated needles and sexual activity after using METH may play an important role in HCV transmission, there is relatively little information available about whether METH directly enhances HCV replication.

METH inhibits immune responses in the liver, facilitating HCV replication in human hepatocytes (Ye et al., 2008). METH inhibits intracellular interferon alpha (IFN-α) expression in human hepatocytes, which is associated with increased HCV replication. In addition, METH compromises the anti-HCV effect of IFN-α. In this regard, METH inhibits the expression of the signal transducer and activator of transcription 1, a key modulator in IFN-mediated responses. METH down-regulates the expression of IFN regulatory factor-5, a crucial transcriptional factor that activates the IFN pathway (Ye et al., 2008). The fact that METH compromises IFN-α-mediated innate immunity against HCV indicates that this drug may have a cofactor role in HCV pathogenesis.

Although less well studied, METH also appears to increase the risk for disease due to hepatitis A virus and hepatitis B virus (HBV) (Gonzales et al., 2008). The factors associated with these infections are similar to that of HCV acquisition. For instance, an outbreak of HBV occurred in a group of METH-abusing individuals sharing injection drug paraphernalia (Vogt et al., 2006). Furthermore, fulminant liver failure due to HBV may be more common in the setting of METH injection (Garfein et al., 2004).

### HIV

There is compelling evidence, although limited in quantity, from both animal and *in vitro* studies that illicit drugs and alcohol directly affect intracellular HIV multiplication, progression to AIDS, and death. Previous research indicated that METH might influence viral entry and integration at the host genome level, promoting HIV production and viremias (Liang et al., 2008; Toussi et al., 2009; Marcondes et al., 2010; Nair and Saiyed, 2011). Specifically, findings suggest an indirect dysregulation of chemokines and costimulatory molecules via DCs, macrophages, and CD4^+^ T lymphocytes, enabling the pathogenesis of HIV.

HIV infection is highly regulated by the expression of the HIV entry co-receptors CXCR4 and CCR5. METH-treated groups demonstrated that both of these receptors exhibited up-regulated expression after METH treatment on dendritic cells, signifying increased susceptibility to HIV infection (Liang et al., 2008; Nair et al., 2009; Nair and Saiyed, 2011). In addition, METH exposure significantly reduced expression of ERK2 and up-regulated p32 MAPK genes. In general, the genes from these signaling pathways govern the regulation of cytokines (IL-2, IL-10, and TNF-α) and if altered, can enhance the production of new HIV virions and deplete CD4+ T cells from the host's immune system (Nair et al., 2009; Nair and Saiyed, 2011). Similarly, METH has demonstrated influence over dopaminergic receptors in previous findings, causing increases in dopamine concentration in extracellular spaces. This excessive accumulation eventually leads to the degeneration of the striatal dopamine terminals and the formation of reactive oxidative stress molecules. In a recent study, D1 and D2 receptors were deleted and METH-treated cells were observed for changes in genetic expression of CCR5 (Nair et al., 2009). Results showed that both D1 and D2 deficient cells reversed the up-regulatory effects of METH on DCs, indicating their involvement in METH-induced HIV infectivity (Reynolds et al., 2007; Nair et al., 2009).

Some factors associated with METH abuse include emotional reasons, social stigmas, depression, heritability, patterns of childhood abuse, and low income (Semple et al., 2008). A growing body of research supports the relationship between METH use and an increase in behaviors (sexual and those related to IDU) that increase risk for HIV infection. Chronic METH use is associated with a 2-fold higher risk of HIV acquisition (Plankey et al., 2007). Among gay and bisexual men, METH is associated with high-risk sexual behavior, HIV infection, and predicts a high incidence of AIDS (Marshall et al., 2011; Nakamura et al., 2011; Lea et al., 2013). In addition to the above-mentioned factors, in a multi-cohort analysis of the LGBT (Lesbian, Gay, Bisexual and Transgender) community, other risk factors for HIV in METH users strongly correlated with young age, IDU, and depression. METH exacerbates HIV pathology, including cognitive deficits, cardiovascular compromise, dental decay, and is strongly suspected to inhibit normal immunological response to secondary infections, such as HCV (Carey et al., 2006; Gonzales et al., 2006; Cruickshank and Dyer, 2009).

HIV infection is associated with progressive CD4^+^ T-cell depletion and immune dysregulation. Direct neurotoxic effects of METH putatively aggravate HIV-associated neuronal injury (Gartner and Liu, 2002; Williams and Hickey, 2002). In addition to CD4^+^ T-lymphocytes, mononuclear phagocytes are primary targets for HIV. HIV-infected macrophages survive for months, actively producing and spreading the virus. METH enhances HIV replication in human macrophages by up-regulation of CCR5 expression, augmenting infectivity and reinforcing the transport of infected leukocytes across the blood brain barrier (Liang et al., 2008). METH administration significantly increases HIV-1 production by both HIV-infected monocytes and CD4^+^ T-lymphocytes *in vitro*. METH increases HIV production and viremia in mice transgenic for a replication-competent HIV provirus and human cyclin T1 (Toussi et al., 2009). Interestingly, METH's interaction with macrophages has illustrated the down-regulation of TLR9 expression, aiding in the HIV infection of these innate cells by mitigating the receptor's antiviral effects (Cen et al., 2013).

METH and HIV-1 appear to cause more neurocognitive deficits than either alone, but their interaction is poorly understood (Rippeth et al., 2004; Cadet and Krasnova, 2007). A transgenic mouse expressing the viral envelope protein gp120 in the CNS has significantly more pronounced stereotypic behavioral responses to METH relative to parental mice, providing *in vivo* evidence that HIV affects the brain's response to the drug (Roberts et al., 2010). Additionally, METH serves as an agonist for the NMDA (N-Methyl-D-aspartate) receptor, activating IDO and COX-2 expression as well as facilitating the eventual production of QUIN, a neurotoxin also induced during HIV infection and can expedite neuronal apoptosis when these mechanisms are combined (Nair and Samikkannu, 2012). Lastly, an evaluation of the impact of METH and Tat on the Wnt/β-catenin signaling pathway, a neuroprotective pathway vital in various CNS functions and negatively regulates HIV-1 replication in astrocytes, revealed that they amplified the inhibitory effect, yet employed individual cascades in an astrocytoma cell line (U87MG) to suppress β-catenin-mediated signaling (Sharma et al., 2011).

HIV pathogenesis can also be enhanced through METH abuse via regulation of members from the signaling lymphocytic activation family (SLAM), which potentially indicates a mechanism by which the drug exacerbates HIV infection (Harms et al., 2012). CD150, a SLAM molecule, was up-regulated on CD4^+^ T cells after METH treatment making these cells susceptible to HIV infection (Harms et al., 2012). METH use enhances HIV neuropathogenesis magnifying the effect of dopamine on HIV infection of macrophages (Gaskill et al., 2009). Although we are just beginning to understand the multifaceted, complex effects of METH in the context of HIV infection, the limited information available suggests that METH facilitates HIV spread, increasing immune cell dysfunction, and exacerbating neuroAIDS.

### Opportunistic fungi

Fungal pathogens have been recently used as empirical models to understand the impact of METH use on host homeostasis and increased permissiveness to opportunistic microorganisms. *Histoplasma capsulatum* is the most prevalent cause of fungal respiratory infections, representing 53.19% of cases of endemic mycoses in the US (Chu et al., 2006). Since *H. capsulatum* is endemic to the Midwestern USA, where METH is a critical public health issue; the fungus is an ideal model organism to study the impact of METH in a systemic disease model. METH abrogates normal macrophage function, resulting in accelerated disease in murine histoplasmosis (Martinez et al., 2009). METH decreases phagocytosis and killing of *H. capsulatum* by primary macrophages. METH exposed *H. capsulatum*-infected mice have increased fungal burdens, increased pulmonary inflammation, and decreased survival. METH exposure results in cytokine dysregulation, aberrant processing of yeasts within macrophages, and immobilization of MAC-1 receptors on the macrophage surface. Additionally, METH inhibits T cell proliferation and alters antibody production, both important components of adaptive immunity. Hence, it is established that METH alters the immune system of a mammalian host, resulting in enhanced disease (Martinez et al., 2009).

The encapsulated fungus *Cryptococcus neoformans* is the most common cause of fungal meningitis in patients with AIDS killing = 600,000 people worldwide (Park et al., 2009). Using a systemic mouse model of infection and *in vitro* assays, it was recently demonstrated that METH stimulates fungal adhesion, capsular polysaccharide release, and biofilm formation in pulmonary tissue (Patel et al., 2013). Interestingly, structural analysis of the capsular polysaccharide of METH-exposed cryptococci revealed that METH alters the carbohydrate composition of this virulence factor, highlighting the fungus's ability to adapt to environmental stimuli, a possible explanation for its pathogenesis. Additionally, METH facilitates *C. neoformans* dissemination from the respiratory tract into the CNS. METH alters BBB integrity and modifies the expression of tight junction and adhesion molecules (Eugenin et al., 2013). These findings provide novel evidence of the impact of METH abuse on the integrity of the cells that comprise the BBB and protect the brain from infection.

## Conclusion and future perspectives

METH use has become increasingly prevalent in recent years, creating a severe public health epidemic and societal burden. The drug adversely changes user behavior, including putting METH users at high risk for the acquisition of diverse infectious diseases. Recent studies have identified a causal linkage between METH and immune dysfunction in mature mammals. METH immunosuppression may underlie the mechanism for the rapid development of AIDS in METH users, progressing from HIV to AIDS within only a few months (CDC, 2007). Investigators are just beginning to decipher the complex effects of METH in the context of HIV infection, but the limited nature of available information suggests that this drug dramatically impacts disease. Understanding the specific mechanisms of METH abuse and HIV will require large epidemiological studies as well as the utilization of relevant animal models that reproduce salient features of HIV infection in humans and are devoid of numerous confounding factors present in human studies.

Another important question yet to be answered is how METH disarms the adaptive immune system, further rendering the host more susceptible to opportunistic infections. We recently showed that the impairment of adaptive immunity by METH diminishes the ability of mammalian hosts to mount and maintain efficient immune responses to pathogens (Martinez et al., 2009). However, the mechanisms responsible for altered regulation of T- and B-cells in METH-exposed hosts require further study. Identification of these underlying mechanisms will highlight new therapeutic and prophylactic methods to improve immunity in the context of drug abuse. These goals are of considerable significance in the fields of immunity, host-pathogen interactions and drug abuse.

There is an urgent need for innovative METH treatment interventions to prevent the acquisition and transmission of infectious diseases. Through utilization of drug abuse treatment and community-based outreach programs, drug abusers can change their HIV risk behaviors (Garfein et al., 2010; Miller et al., 2010; Naar-King et al., 2010). Through targeted outreach and awareness programs, the prevalence of drug abuse and drug-related risk behaviors, such as needle-sharing and unsafe sexual practices, can be reduced significantly, thus decreasing the risk of disease acquisition. This is a challenge because due to recent reduction in healthcare funding usually compromises the viability of these preventive programs. Healthcare providers should be trained to recognize signs of METH addiction, and work openly and honestly with their patients to address the detrimental effects of METH addiction.

At this time, cognitive behavioral and contingency management interventions are the most effective treatments for METH addiction (Rawson et al., 2004; Roll et al., 2006). For example, the Matrix Model is a comprehensive behavioral treatment approach for the reduction of METH abuse that merges cognitive therapy, drug testing, family education, 12-Step support, individual counseling and reinforcement for nondrug-related activities (Rawson et al., 2004). Contingency management interventions also offer tangible incentives in exchange for participating in therapy and sustaining abstinence. (Roll et al., 2006) Currently, no specific medications exist that counteract the effects of METH or that prolong abstinence from the abuse of METH by an addict. However, novel anti-METH immunotherapies, primarily in the form of monoclonal antibodies and lipid-based vaccines, are in early clinical trial phases and act as pharmacokinetic antagonists, isolating METH and its metabolites from vulnerable areas in the brain and minimizing the toxic effects of the drug (Peterson et al., 2013; Rüedi-Bettschen et al., 2013; Collins et al., 2014; Hambuchen et al., 2014).

Finally, the research described to date is likely to be only the tip of the proverbial iceberg, such that numerous other diseases, especially infectious diseases, are likely to be significantly modified by METH. The propagation of this disease, along with many other viral and bacterial contagions, demonstrates the necessity for continued studies in this area of healthcare and substance abuse. Until the use of METH is strictly curtailed, the impact of METH on our society will continue to be severe.

### Conflict of interest statement

The authors declare that the research was conducted in the absence of any commercial or financial relationships that could be construed as a potential conflict of interest.
